# 

**Published:** 1898-07

**Authors:** 


### CONTENTS.

**ORIGINAL COMMUNICATIONS.** PAGE

Secrets and Patents *versus* Professional Progress. By J. MORGAN HOWE, M.D.S., M.D., New York . . . . . 413
What should be the Character and Policy of Dental Journalism? By LOUIS JACK, D.D.S., Philadelphia . . . . . 426
Independent Journalism. By WILLIAM H. POTTER, D.M.D., Boston, Mass. . . . 433
Professional Atmosphere and Morals. By B. HOLLY SMITH, M.D., D.D.S., Baltimore, Md. . . . . . 439

**ABSTRACTS AND TRANSLATIONS.**

The Relative Efficiency of Various Anæsthetics . . . . . 445

**REPORTS OF SOCIETY MEETINGS.**

The New York Institute of Stomatology . . . . . 448

**EDITORIAL.**

The Divorcement of Dental Literature from Trade . . . . . 482
The National Dental Association . . . . . 486
The Funeral of Dr. Thomas W. Evans . . . . . 487

**BIBLIOGRAPHY** . . . . . 488

**NOTES AND COMMENTS** . . . . . 490

**CURRENT NEWS.**

Alumni Society of the Philadelphia Dental College . . . . . 491
Central Dental Association of Northern New Jersey . . . . . 492
Chicago Dental Society . . . . . 492
Dental Society of the State of New York . . . . . 492

